# Comparison of real-world healthcare resource utilization and costs among patients with hereditary angioedema on lanadelumab or berotralstat long-term prophylaxis

**DOI:** 10.57264/cer-2024-0205

**Published:** 2025-02-20

**Authors:** Nicole Princic, Kristin A Evans, Chintal H Shah, Krystal Sing, Salomé Juethner, Bob G Schultz

**Affiliations:** 1 Merative, Ann Arbor, MI 48108, USA; 2 University of Maryland, Baltimore, MD 21201, USA; 3 Takeda Pharmaceuticals USA, Inc., Lexington, MA 02421, USA

**Keywords:** berotralstat, healthcare costs, healthcare resource utilization, hereditary angioedema, lanadelumab, long-term prophylaxis

## Abstract

**Aim:**

Hereditary angioedema (HAE) is a rare and chronic genetic condition. Lanadelumab and berotralstat, two plasma kallikrein inhibitors, have both been approved for long-term prophylaxis in patients with HAE; however, real-world data comparing costs and healthcare resource utilization (HCRU) are lacking.

**Materials & methods:**

This retrospective study used administrative healthcare insurance claims data (Merative™ MarketScan^®^ Commercial, Medicare and Early View Research Databases; 1 July 2017–31 July 2023) to identify patients with HAE who initiated lanadelumab or berotralstat and were persistent for ≥18 months or 6 months, respectively. Sex, baseline healthcare costs and baseline number of on-demand treatment/short-term prophylaxis medication claims were used to calculate covariate balancing propensity scores for inverse probability of treatment weighting. Following weighting, outcomes during the 6-month follow-up period in patients receiving berotralstat were compared with those during months 0–6, 7–12 and 13–18 in lanadelumab-treated patients.

**Results:**

Fifty-seven lanadelumab- and 32 berotralstat-treated patients were included. After weighting, more berotralstat-treated patients had an all-cause inpatient admission (berotralstat, 9.4%; lanadelumab, months 0–6, 4.0%, 7–12, 1.8%, months 13–18, 2.0%) and emergency room visit (berotralstat, 21.9%; lanadelumab, months 0–6, 14.0%, 7–12, 8.0%, months 13–18, 17.9%). Total HAE treatment costs were similar during months 0–6 (lanadelumab, $377,326 vs berotralstat, $373,010), but decreased in months 7–12 ($319,967) and 13–18 ($283,241) of lanadelumab. On-demand treatment/short-term prophylaxis costs were lower for lanadelumab across the three follow-up periods than for berotralstat during months 0–6 (berotralstat, $60,451; lanadelumab, months 0–6, $46,336, months 7–12, $37,578, months 13–18, $23,968). The proportion of lanadelumab-treated patients who reduced dosing frequency was 24.8% during months 7–12 and 21.6% during months 13–18.

**Conclusion:**

Patients with HAE initiating lanadelumab versus berotralstat may require less on-demand and supportive HAE treatments and incur lower treatment-related and total healthcare costs. The ability to reduce lanadelumab dosing frequency after an attack-free period may be key in treatment selection, given the combination of cost savings and lower healthcare resource utilization.

## Plain language summary: differences in the use of services & healthcare costs in patients with hereditary angioedema treated with lanadelumab or berotralstat

**What is this article about?:**

When patients submit a claim to their health insurance provider, details are recorded for real-world medical research. This study used insurance databases to assess costs and use of healthcare services for patients taking lanadelumab or berotralstat, which are attack-prevention treatments for hereditary angioedema.

**What were the results?:**

Results showed that patients taking lanadelumab needed less short-term and supportive hereditary angioedema treatments and had fewer hospitalizations and emergency room visits. Treatment and healthcare costs were cheaper for lanadelumab than for berotralstat.

## Graphical abstract

**Figure:**
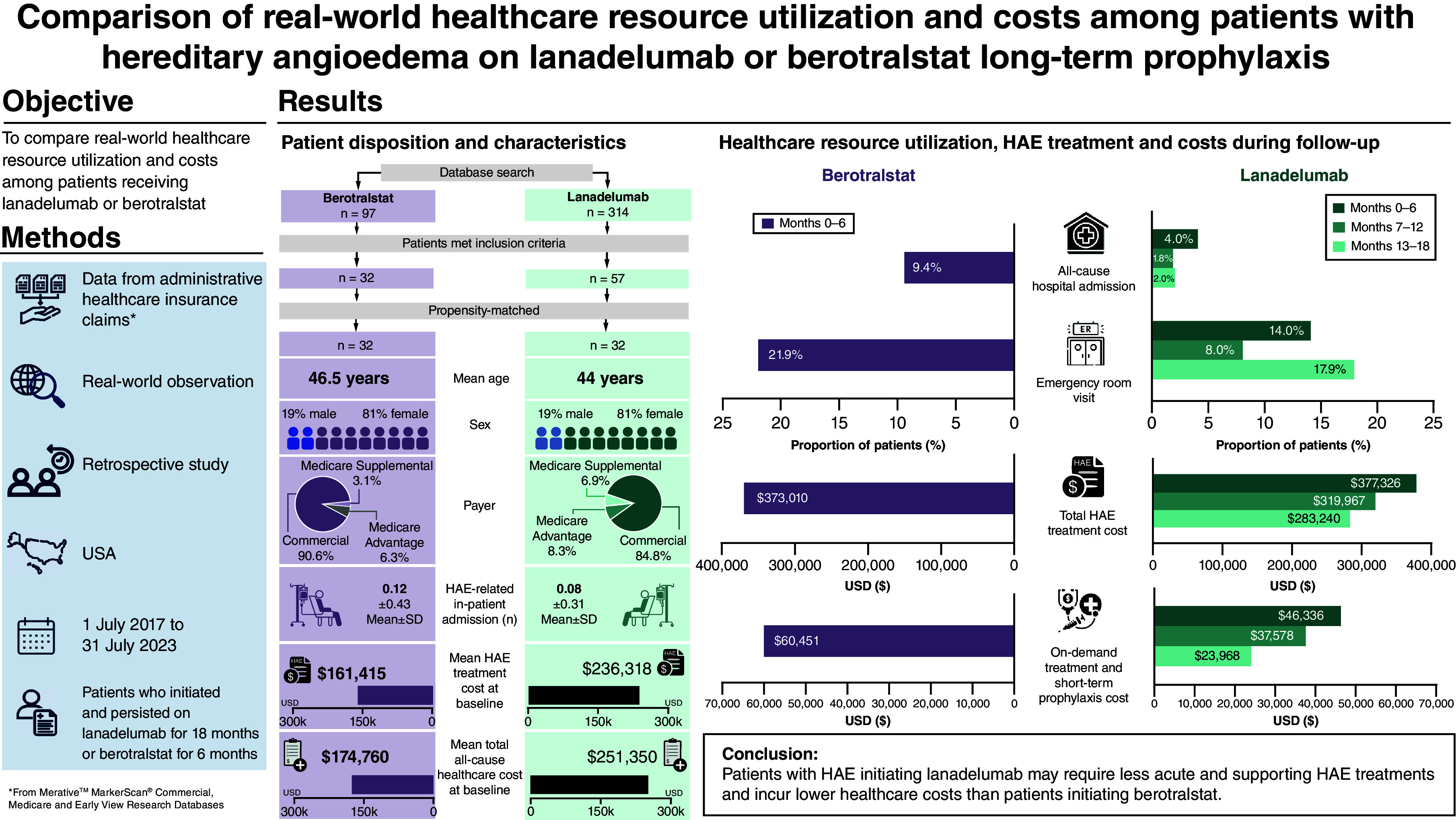

## Background

Hereditary angioedema (HAE) is a rare, incurable and chronic genetic condition [1–3]. It manifests as spontaneous and unpredictable episodes of cutaneous or submucosal swelling that can affect the face, upper or lower extremities, genitals, abdominal organs and the upper airway [1–3]. HAE attacks are disfiguring and painful, and laryngeal attacks can be fatal due to asphyxiation [1,2]. In addition, the unpredictability and variability of the clinical presentation and symptoms of HAE attacks make them difficult to characterize and manage effectively [4,5].

Treatment for HAE often takes a multipronged approach, including on-demand treatment (ODT) for acute HAE attacks, short-term prophylaxis (STP) for recognized triggers such as dental work, surgery or stressful life events, and long-term prophylaxis (LTP) [6]. Management of HAE using LTP has the potential to reduce the burden of the disease by preventing HAE attacks and normalizing a patient’s life [1,7]. Two plasma kallikrein inhibitors have been approved by the US FDA in recent years: lanadelumab in August 2018 and berotralstat in December 2020 [8,9]. Lanadelumab and berotralstat have both demonstrated efficacy and safety in phase III clinical trials [10–12]. The use of lanadelumab has also been shown to reduce serious HAE attacks and the need for ODT [10]. Although data from head-to-head clinical trials are not available, an indirect treatment comparison has demonstrated a more significant reduction in the HAE attack rate with lanadelumab versus berotralstat [13]. Due to the high burden of disease and the associated costs, payers are interested in how to optimally manage the HAE patient population [14].

Patients treated with subcutaneous lanadelumab (300 mg dose) every 2 weeks (Q2W) can reduce their dose to once every 4 weeks (Q4W), per the labeled indication, if HAE attacks are well-controlled [8]. Berotralstat (150 mg oral capsule taken once-daily) only has a dose reduction specified in its product labeling for patients with hepatic impairment or persistent gastrointestinal reactions (110 mg), not for those whose HAE is well-controlled [9]. To put any potential cost savings associated with a reduction in lanadelumab dose frequency into context, real-world data are needed to describe healthcare resource utilization (HCRU), healthcare costs and HAE treatment patterns among patients persistent on berotralstat LTP.

The objective of the current analysis is to describe the demographics, baseline clinical characteristics, treatment patterns, HCRU and costs among patients who were either persistently prescribed berotralstat for 6 months or lanadelumab for 18 months.

## Materials & methods

### Study design & population

This retrospective, observational study used administrative healthcare insurance claims data from the Merative™ MarketScan^®^ Commercial, Medicare and Early View Research Databases between 1 July 2017 and 31 July 2023 to identify patients with HAE in the US who initiated lanadelumab or berotralstat and were persistent on those treatments for ≥18 months or 6 months, respectively. Different observation periods for lanadelumab and berotralstat were chosen in order to preserve sample size given the lack of long-term real-world data among patients treated with berotralstat owing to its more recent approval, because the costs of berotralstat are not expected to change substantially after 6 months as there is no indication for reduced dosing frequency, and that the steady state of berotralstat is reached by day 6 to 12.

Persistence on lanadelumab was defined as having no more than a 60-day gap in days of supply during the 18-month follow-up period. Lanadelumab is usually dispensed as a 28-day supply, allowing for Q2W dosing. However, given that the lanadelumab label indicates that administration frequency can be reduced to Q4W, it is possible that a 28-day supply can cover 56 days. Therefore, a 60-day gap in days of supply was selected to include patients with Q4W dosing, while also minimizing the inclusion of patients who were not persistent during the study period. This methodology has been published in previous studies [15]. Persistence on berotralstat was defined as having no more than a 30-day gap in days of supply during the 6-month follow-up period considering there is no labeled indication for dosing intervals other than once daily.

Patients who initiated lanadelumab treatment were included in the study if they had at least one medical claim with the Healthcare Common Procedure Coding System code or ≥1 pharmacy claim with the National Drug Code (NDC) for lanadelumab between 1 January 2018 and 31 July 2023; at least 6 months of continuous insurance enrollment with medical and pharmacy benefits before the index date (baseline period); at least 18 months of continuous insurance enrollment with medical and pharmacy benefits after and including the index date (follow-up period); and no claims for lanadelumab during the baseline period (new users) nor claims for berotralstat during the entire study period. Patients who initiated berotralstat were included in the study if they had at least one medical claim with an NDC for berotralstat between 1 December 2020 and 31 July 2023; at least 6 months of continuous insurance enrollment with medical and pharmacy benefits before the index date (baseline period); at least 6 months of continuous insurance enrollment with medical and pharmacy benefits after and including the index date (follow-up period); and no claims for berotralstat during the baseline period. For both treatments, the earliest claim was considered the index date. The different inclusion criteria for patients who initiated lanadelumab or berotralstat treatment were due to differences in the approval dates (August 2018 for lanadelumab and December 2020 for berotralstat) and dosing of the two treatments. Unlike lanadelumab, berotralstat does not have a labeled indication to reduce dose after 6 months, and therefore the costs of berotralstat are not expected to change substantially.

An overview of the study design, key study periods and data assessed are shown in Supplementary Figure 1. Demographic characteristics were assessed on the index date for both cohorts, clinical characteristics were assessed during the 6-month baseline period for both cohorts, and HCRU and cost outcomes were assessed during the 6-month baseline period for both cohorts, during the 6-month follow-up period for the berotralstat cohort and during three intervals of the 18-month follow-up period for the lanadelumab cohort: months 0–6, months 7–12 and months 13–18.

### Demographic & clinical characteristics & HAE-related healthcare encounters

Demographic characteristics included age, sex, payer, plan type and index year (2018–2023). Clinical characteristics included the Deyo Charlson Comorbidity Index (DCI), individual DCI comorbidities and comorbid inflammatory bowel disease, triggers of an HAE attack and/or common comorbid conditions of patients with HAE and warning signs and symptoms of HAE attacks. HAE-related healthcare encounters were assessed by the presence of the International Classification of Diseases, 10th Revision code D84.1, which is a nonspecific code for defects in the complement system that includes HAE. Data include overall healthcare encounters and encounters categorized according to setting, including inpatient admissions, emergency room (ER) visits, outpatient office visits and other (nonoffice) outpatient visits.

### HCRU & costs

All-cause HCRU and costs during the baseline and follow-up periods included the number of visits and costs for each of inpatient admissions, ER visits, outpatient physician office visits and other outpatient services. For outpatient pharmacy prescriptions, the number of prescriptions and costs were collected. Total costs comprised medical service costs (inpatient admissions and outpatient visits) plus outpatient pharmacy costs.

HAE treatment use and costs during the baseline and follow-up periods included the number of claims and costs for ODT/STP treatment, supportive care treatment (namely opioids, nonsteroidal anti-inflammatory drugs and antiemetics) and other LTP medications (subcutaneous or intravenous C1 esterase inhibitors, berotralstat and lanadelumab). Outlier ODT/STP treatment claims were considered those that had paid amounts greater than $200,000 (>95th percentile of the distribution). The cost of these outlier claims was imputed with the median amount paid among claims with the same NDC that were less than $200,000. Total HAE treatment cost during the baseline period included both outpatient prescription and physician-administered drugs and comprised costs for ODT/STP treatment, supportive care treatment, other LTP treatments and, among patients in the berotralstat cohort only, lanadelumab. Total HAE treatment cost during the follow-up period included costs for ODT/STP treatment, supportive care treatment, berotralstat, lanadelumab and other LTP treatments. Lanadelumab-treated patients were also assessed for evidence of reduced dosing frequency during months 7–12 and 13–18, defined as a decrease in lanadelumab costs of ≥25% compared with months 0–6. In order to account for fluctuations in costs owing to inflation and other market factors, all dollar estimates were inflated to 2022 dollars using the medical care component of the Consumer Price Index.

### Data analysis

Descriptive statistics were reported for all aforementioned variables and were compared between patients prescribed lanadelumab and berotralstat. Continuous variables were reported as means and standard deviations (SD), and categorical variables as counts and percentages. Patient sex, baseline total healthcare costs and baseline number of ODT/STP medication claims were used to calculate covariate balancing propensity scores (CBPS) [16] for each patient. Due to sample size considerations, the regression models used to derive CBPS were limited to these few key variables. However, the variables that were selected were assumed to have a relevant association with the outcomes of interest. The CBPS for each patient represented their probability of receiving berotralstat versus lanadelumab, given their unique set of characteristics. Data for lanadelumab patients were then weighted by the inverse of their CBPS (inverse probability of treatment weighting), maximizing the balance between groups based on the variables included in the propensity score model. Outcomes during the 6-month follow-up period in patients receiving berotralstat were compared with those during each of the three 6-month intervals for lanadelumab-treated patients.

While this research is descriptive, statistical significance was tested to further understand any differences in HCRU and costs between patients receiving lanadelumab versus berotralstat. Observed differences in HCRU and costs between the weighted lanadelumab and berotralstat cohorts were compared using *t*-tests with Huber–White standard errors for continuous variables, and Chi-square tests with second order Rao–Scott corrections for categorical variables. Data were extracted and analyzed using SAS software, version 9.4 (SAS Institute, NC, USA).

## Results

### Study population

Of the 314 patients who had at least one claim for lanadelumab between 1 January 2018 and 31 July 2023, 57 met all inclusion criteria (Supplementary Table 1). Of the 97 patients who had at least one claim for berotralstat between 1 December 2020 and 31 July 2023, 32 met all inclusion criteria (Supplementary Table 1). The demographic characteristics of patients treated with berotralstat and lanadelumab pre- and post-weighting are shown in Table 1. For lanadelumab patients, the post-weighting effective sample size (n = 32) represents the sum of the inverse probability of treatment weights for all 57 patients. Weighted standardized differences (STD) indicate that the two cohorts were well-balanced by sex, and the balance between other characteristics, including age and index year, was improved post-weighting. The unweighted baseline comorbidity burden was higher among lanadelumab- than berotralstat-treated patients (Supplementary Table 2), as indicated by the higher mean ± SD DCI score (0.86 ± 1.82 vs 0.53 ± 0.84; STD, 0.23). The DCI scores were well-balanced between the cohorts after weighting (STD, 0.06).

**Table 1.** Baseline demographic characteristics and hereditary angioedema-related healthcare resource utilization and treatment utilization during baseline.

Characteristics	Berotralstat (n = 32)	Lanadelumab unweighted (n = 57)	STD	Lanadelumab weighted (n = 32)^†^	STD
Age, years					
Mean ± SD	46.5 ± 17.0	43.2 ± 15.6	0.20	44.0 ± 13.8	0.15
Median (min, max)	46.5 (12.0, 88.0)	41.0 (15.0, 87.0)		43.0 (16.0, 87.0)	
Sex, n (%)					
Female	26 (81.3)	24 (42.1)	0.52	25.7 (81.1)	0.00
Male	6 (18.7)	33 (57.9)	0.52	6.0 (18.9)	0.00
Payer, n (%)					
Commercial	29 (90.6)	54 (94.7)	0.16	26.9 (84.8)	0.20
Medicare Supplemental	1 (3.1)	2 (3.5)	0.02	2.2 (6.9)	0.15
Medicare Advantage	2 (6.3)	1 (1.8)	0.23	2.6 (8.3)	0.23
Insurance plan type, n (%)					
Comprehensive/indemnity	2 (6.3)	2 (3.5)	0.13	0.9 (2.7)	0.15
EPO/PPO	15 (46.9)	26 (45.6)	0.03	13.7 (43.3)	0.07
POS/POS with capitation	0 (0.0)	6 (10.5)	0.49	2.7 (8.4)	0.34
HMO	7 (21.9)	9 (15.8)	0.16	5.5 (17.4)	0.11
CDHP/HDHP	8 (25.0)	13 (22.8)	0.05	8.3 (26.1)	0.03
Other/unknown	0 (0.0)	1 (1.8)	0.19	0.7 (2.2)	0.21
Index year, n (%)					
2018	0 (0.0)	12 (21.1)	0.73	5.7 (18.1)	0.53
2019	0 (0.0)	25 (43.9)	1.25	11.7 (36.9)	0.82
2020	0 (0.0)	10 (17.5)	0.65	7.9 (25.0)	0.79
2021	11 (34.4)	9 (15.8)	0.44	6.1 (19.3)	0.32
2022	20 (62.5)	1 (1.8)	1.71	0.2 (0.7)	1.28
2023	1 (3.1)	0 (0.0)	0.25	0.0 (0.0)	0.18
Any HAE-related healthcare visit^‡^, n (%)	26 (81.3)	42 (73.7)	0.18	23.9 (75.5)	0.15
HAE-related inpatient admissions, n, mean ± SD	0.12 ± 0.43	0.10 ± 0.37	0.05	0.08 ± 0.31	0.08
Average length of inpatient admission, mean ± SD (months)	5.3 ± 3.2	2.2 ± 2.0	1.16	1.5 ± 1.8	1.19
HAE-related ER visits^§^, n, mean ± SD	0.12 ± 0.33	0.31 ± 1.85	0.15	0.13 ± 1.25	0.04
HAE-related non-ER outpatient visits^§^, n, mean ± SD	3.0 ± 3.4	5.6 ± 11.8	0.30	5.0 ± 11.8	0.60
ODT/STP treatment, n (%)	19 (59.4)	39 (68.4)	0.19	16.9 (53.3)	0.12
ODT/STP treatment claims among all patients, n, mean ± SD	1.9 ± 2.8	4.4 ± 6.6	0.49	1.9 ± 2.9	0.01
Supportive care treatments,^¶^ n (%)	16 (50.0)	16 (28.1)	0.46	6.6 (20.8)	0.58
Supportive care treatment claims among all patients, n, mean ± SD	1.7 ± 3.3	0.9 ± 2.5	0.26	0.5 ± 1.7	0.36
Other LTP treatments, n (%)					
Intravenous C1 esterase inhibitor	1 (3.1)	10 (17.5)	0.83	5.2 (16.5)	0.77
Subcutaneous C1 esterase inhibitor	2 (6.3)	13 (22.8)	0.48	7.8 (24.5)	0.76
Lanadelumab	5 (15.6)	0 (0.0)	0.61	0.0 (0.0)	0.43

† Patients’ data were weighted by the inverse of their covariate balanced propensity score; post-weighting sample sizes are the sums of patients’ inverse probability of treatment weights and therefore are not whole numbers for the lanadelumab cohort.

‡ Inpatient admissions, ER visits and other non-ER outpatient claims containing ICD-10-CM diagnosis code D84.1 (defects in the complement system).

§ If a patient had an inpatient admission and an ER visit or non-ER outpatient visit with an HAE diagnosis on the same date, only the inpatient admission was counted. If they had an ER visit and a non-ER outpatient visit with an HAE diagnosis on the same date (and no inpatient admission), only the ER visit was counted.

¶ Supportive care treatments included opioids, nonsteroidal anti-inflammatory drugs and antiemetics.

CDHP: Consumer-driven health plan; EPO: Exclusive provider organization; ER: Emergency room; HAE: Hereditary angioedema; HCRU: Healthcare resource use; HDHP: High-deductible health plan; HMO: Health maintenance organization; ICD-10: International Classification of Diseases, Tenth Revision; LTP: Long-term prophylaxis; ODT: On-demand treatment; POS: Point of service; PPO: Preferred provider organization; SD: Standard deviation; STD: Standardized difference; STP: Short-term prophylaxis.

### Baseline HAE treatment, HCRU & costs

Compared with the berotralstat cohort, a higher proportion of patients in the lanadelumab cohort used ODT/STP treatments for HAE, and they had a higher number of ODT/STP claims per patient during the baseline period (Table 1). Conversely, compared with the berotralstat cohort, a lower proportion of patients in the lanadelumab cohort used supportive care treatments, with less baseline supportive care treatment claims per patient.

A higher proportion of patients in the berotralstat cohort than the lanadelumab cohort had an HAE-related healthcare encounter at baseline (Table 1). The number of HAE-related hospital admissions was similar between the cohorts; however, patients in the berotralstat group had longer average HAE-related inpatient stays (5.3 vs 2.2 days for the lanadelumab cohort). In addition, patients in the lanadelumab cohort had more HAE-related outpatient visits (both ER and non-ER) than those in the berotralstat cohort.

Data for all-cause HCRU during baseline are provided in Supplementary Table 3. The proportion of patients with at least one all-cause hospital admission was similar between treatment cohorts, although the number of all-cause hospital admissions was slightly higher in berotralstat- than lanadelumab-treated patients. The proportion of berotralstat-treated patients with at least one ER visit was higher than that of lanadelumab-treated patients, whereas the mean number of ER visits was higher in the lanadelumab cohort.

HAE treatment costs at baseline were higher for the lanadelumab cohort than the berotralstat cohort during the baseline period ($236,318 vs $161,415). ODT/STP treatment costs contributed to 59.8% of baseline HAE treatment costs ($141,252) in lanadelumab-treated patients and 48.4% of baseline treatment costs ($78,121) in berotralstat-treated patients. LTP treatments contributed 39.5% ($93,463) and 51.0% ($82,380) of baseline treatment costs in the lanadelumab and berotralstat cohorts, respectively.

Total all-cause healthcare costs were 44% greater in the lanadelumab cohort than the berotralstat cohort during the baseline period ($251,350 vs $174,760). Baseline ER costs were 51% greater in the lanadelumab cohort compared with the berotralstat cohort ($1151 vs $761). Outpatient pharmacy costs contributed to over 90% of total healthcare costs during the baseline period for both treatment cohorts, although they were 40% higher in the lanadelumab cohort than in the berotralstat cohort ($229,645 vs $163,849).

After weighting, the baseline treatment claims, HCRU and costs were well-balanced between lanadelumab- and berotralstat-treated patients.

### HCRU, HAE treatment & costs during follow-up

**Figure 1.**
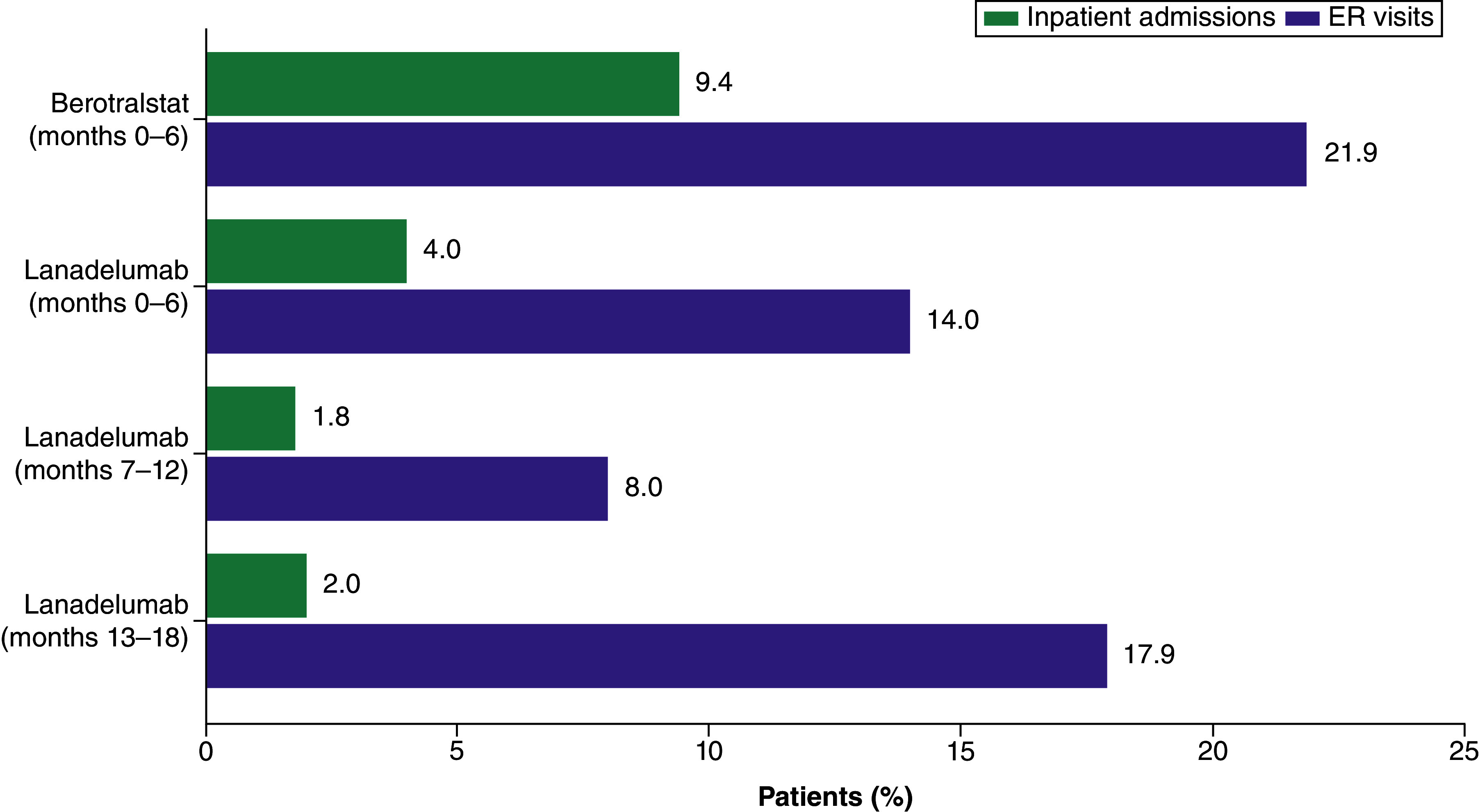
Proportion of patients with inpatient admissions and emergency room visits during follow-up. ER: Emergency room.

**Figure 2.**
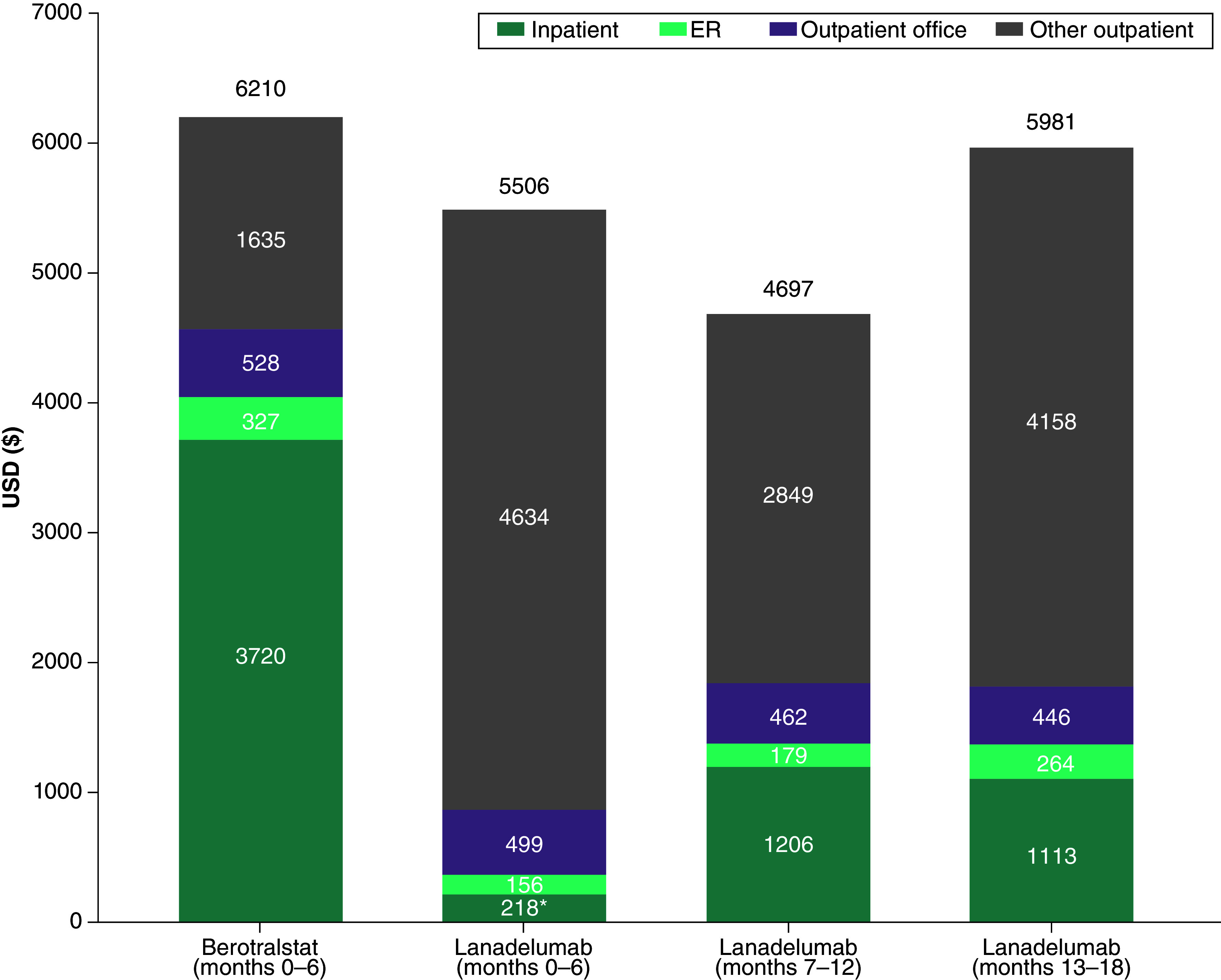
All-cause medical service costs during follow-up. *p < 0.05 versus berotralstat months 0–6. ER: Emergency room.

In the follow-up period after index treatment initiation, a higher proportion of berotralstat-treated patients had at least one all-cause hospital admission at months 0–6 than lanadelumab-treated patients during months 0–6, 7–12 or 13–18 (Figure 1). The mean ± SD number of all-cause admissions for patients treated with berotralstat during the first 6 months of follow-up was 0.13 ± 0.42, and was 0.04 ± 0.20, 0.02 ± 0.13 and 0.02 ± 0.14 for lanadelumab-treated patients during months 0–6, 7–12 and 13–18, respectively. All-cause inpatient costs were 94% (p < 0.05), 68% and 70% lower among lanadelumab-treated patients during the three follow-up intervals, compared with inpatient costs during months 0–6 among berotralstat-treated patients (Figure 2).

Similarly, a higher proportion of berotralstat-treated patients had an all-cause ER visit at months 0–6 than lanadelumab-treated patients during months 0–6, 7–12 or 13–18 (Figure 1). The mean ± SD number of ER visits for patients treated with berotralstat during months 0–6 was 0.84 ± 2.23 and was 0.34 ± 1.03, 0.26 ± 1.25 and 0.45 ± 1.13 for patients treated with lanadelumab during months 0–6, 7–12 and 13–18, respectively. All-cause ER costs were approximately 50% lower in lanadelumab-treated patients than in berotralstat-treated patients during months 0–6, and remained lower during subsequent follow-up intervals (Figure 2).

During the 6-month follow-up period of berotralstat-treated patients, the costs of ODT/STP treatments were $60,451. During the months 0–6, 7–12 and 13–18 follow-up periods in lanadelumab-treated patients, the costs for ODT/STP treatments were $46,336, $37,578 and $23,968, respectively. The proportion of patients using ODT/STP treatments was higher among lanadelumab-treated patients than berotralstat-treated patients (Table 2). However, the proportion of lanadelumab-treated patients using ODT/STP treatment decreased over the subsequent follow-up intervals and was lower during months 7–12 and 13–18, compared with berotralstat-treated patients during months 0–6. The mean number of ODT/STP claims was lower for patients receiving lanadelumab in all three follow-up intervals, compared with those receiving berotralstat during months 0–6 (Table 2).

**Table 2.** Hereditary angioedema treatment utilization during follow-up (weighted analysis^†^).

	Berotralstat (n = 32)	Lanadelumab (n = 32)		
	Months 0–6	Months 0–6	Months 7–12	Months 13–18
ODT/STP treatment, n (%)	17 (53.1)	19.6 (61.7)	12.2 (38.6)	14.4 (45.6)
ODT/STP treatment claims among all patients, n, mean ± SD	1.7 ± 2.4	1.4 ± 2.5	1.1 ± 2.5	1.0 ± 2.2
Supportive care treatments,^‡^ n (%)	6 (18.8)	4.5 (14.1)	5.2 (16.3)	6.6 (20.9)
Supportive care treatment claims among all patients, n, mean ± SD	1.0 ± 2.9	0.3 ± 1.1	0.4 ± 1.3	0.5 ± 1.4
Other LTP treatments, n (%)				
Intravenous C1 esterase inhibitor	1 (3.1)	0.1 (0.2)	0.0 (0.0)	0.0 (0.0)
Subcutaneous C1 esterase inhibitor	0 (0.0)	0.6 (2.0)	0.2 (0.6)	0.0 (0.0)
Lanadelumab	1 (3.1)	31.7 (100.0)	31.7 (100.0)	31.7 (100.0)
Lanadelumab dose frequency reduction, n (%)	–	–	7.9 (24.8)	6.9 (21.6)

† Patients’ data were weighted by the inverse of their covariate balanced propensity score (CBPS); post-weighting sample sizes are sums of patients’ inverse probability of treatment weights and are therefore not whole numbers for the lanadelumab cohort.

‡ Supportive care treatments include opioids, nonsteroidal anti-inflammatory drugs and antiemetics.

CBPS: Covariate balancing propensity score; HAE: Hereditary angioedema; LTP: Long-term prophylaxis; ODT: On-demand treatment; SD: Standard deviation; STP: Short-term prophylaxis.

The proportion of patients with a claim for supportive care treatments was lower in the first two lanadelumab follow-up periods compared with months 0–6 of berotralstat treatment but was slightly higher in months 13–18 of lanadelumab treatment. The mean number of claims for supportive care treatments was lower among lanadelumab-treated patients during each follow-up interval compared with berotralstat-treated patients during months 0–6. There was little use of LTP treatments other than the index drug during follow-up; one patient treated with berotralstat had evidence of lanadelumab use, and ≤2.0% of lanadelumab-treated patients had evidence of subcutaneous C1 esterase inhibitor use during follow-up. Among lanadelumab-treated patients, 24.8% had a reduction in dosing frequency during months 7–12 and 21.6% had a reduction during months 13–18. Pharmacy costs were similar between berotralstat-treated and lanadelumab-treated patients during the first 6 months of follow-up ($378,093 and $377,056, respectively), but were 15% ($321,353) and 25% ($283,926) lower among lanadelumab-treated patients during months 7–12 and 13–18, respectively.

**Figure 3.**
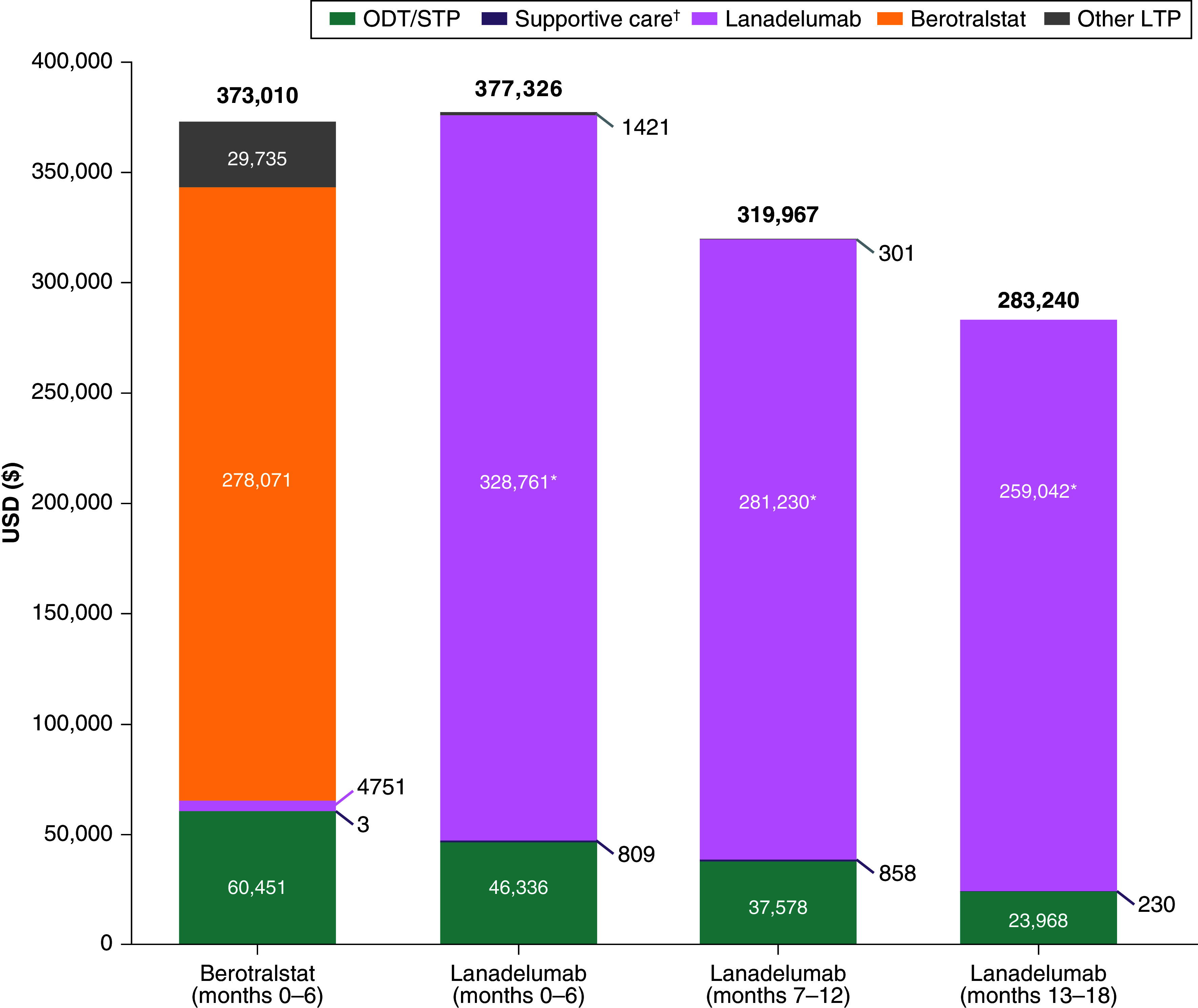
Hereditary angioedema treatment costs during follow-up. *p < 0.05 versus berotralstat months 0–6. †Supportive care includes opioids, nonsteroidal anti-inflammatory drugs and antiemetics. HAE: Hereditary angioedema; LTP: Long-term prophylaxis; ODT: On-demand treatment; STP: Short-term prophylaxis.

Total all-cause healthcare costs (medical costs and outpatient pharmacy costs) for patients treated with berotralstat during months 0–6 were $384,302 and were $382,562, $326,049 and $289,907 for lanadelumab-treated patients during months 0–6, 7–12 and 13–18, respectively (Supplementary Figure 2). Compared with months 0–6 for the berotralstat-treated patients, total healthcare costs were 15% lower among lanadelumab-treated patients during months 7–12 and 25% lower during months 13–18. The total costs for HAE treatments were similar between treatments during months 0–6, but decreased in subsequent lanadelumab follow-up periods (Figure 3). Total treatment costs were 14.2% lower among lanadelumab-treated patients during months 7–12, when compared with the first 6 months of berotralstat treatment. This difference increased to 24.1% during months 13–18 of lanadelumab treatment.

## Discussion

This retrospective analysis using administrative healthcare insurance claims data from the Merative™ MarketScan^®^ databases described the characteristics as well as the all-cause and HAE treatment–related HCRU and costs of patients who initiated, and were persistent on, berotralstat or lanadelumab LTP. Compared with patients who initiated berotralstat, patients who initiated lanadelumab were slightly younger, were more likely to be male and had a greater overall comorbidity burden. In the 6 months prior to initiation of the index treatment, patients in the lanadelumab cohort had a greater baseline burden of HAE, including more HAE-related outpatient visits and more claims for ODT/STP HAE treatments than those in the berotralstat cohort.

Post treatment initiation, and after accounting for baseline differences with weighted analysis, patients treated with lanadelumab had lower utilization of all healthcare services compared with patients treated with berotralstat, including fewer inpatient admissions, ER visits, office visits and fewer prescription drug claims. Patients treated with lanadelumab also had evidence of lower HAE disease activity following treatment initiation, with fewer claims for ODT/STP and supportive care treatments during each of the three 6-month follow-up intervals, compared with the first 6 months of berotralstat treatment. These differences in HCRU amounted to 25% lower all-cause healthcare costs and 24% lower HAE treatment costs during months 13–18 of lanadelumab persistence, compared with 6 months of persistent berotralstat treatment. The evidence of reduced dosing frequency in nearly a quarter of patients during months 7–12 and 13–18 of lanadelumab treatment suggests that the lower costs over time are at least partly attributable to the labeled indication for a dose frequency reduction in well-controlled patients after 6 months.

These data are consistent with previous US-based, retrospective cohort studies that have used administrative claims data (MarketScan) to investigate trends in costs over time among patients with HAE who received LTP with lanadelumab [15,17]. The first study investigated lanadelumab costs over time in patients with HAE (n = 54) who received persistent treatment over 18 months, as well as treatment patterns such as reduced dosing frequency in appropriate patients [15]. As in this study, lanadelumab- and HAE-specific costs were assessed during three follow-up periods (0–6, 7–12 and 13–18 months). Reduced dosing frequency was defined as a ≥25% decrease in lanadelumab costs from months 0–6 to months 7–12 or 13–18. HAE treatment costs decreased over time from $377,076 in months 0–6, to $329,855 in months 7–12 and $286,074 in months 13–18, with downward cost trends for ODT/STP HAE medications contributing to the lower total HAE treatment costs over time. Additionally, reduced dosing frequency of lanadelumab was reported for 25 patients (46.3%); 15 (27.8%) during months 7–12 and 10 (18.5%) during months 13–18. Most (n = 13) of the patients who reduced dosing frequency during months 7–12 had evidence of remaining at that dosing frequency during months 13–18, thus suggesting being well-controlled. Compared with those with no evidence of reduced dosing frequency, patients who were well-controlled after dose frequency reduction were slightly younger (mean age = 39.8 vs 40.3 years), more likely to be female (69 vs 62%), had a lower comorbidity burden (mean DCI score = 0.75 vs 0.83) and had a lower frequency of most HAE attack signs and symptoms and fewer HAE-related inpatient admissions and ER visits prior to lanadelumab initiation (data not published). A further real-world study also reported that the efficacy of lanadelumab was maintained in patients whose lanadelumab dosing interval was gradually increased once they were symptom free, including dosing intervals not within the lanadelumab label [18]. It is possible that some patients receiving lanadelumab included in this analysis had a reduced dosing frequency even greater than every 4 weeks.

The second study investigated the HCRU, costs and treatment patterns in new users of lanadelumab (n = 47) and subcutaneous esterase inhibitors (n = 38) from 180 days prior to and 1 year after treatment initiation [17]. Annualized HAE-related ER visits and hospitalizations decreased after treatment initiation from 1.8 to 0.6 for patients on lanadelumab and from 1.3 to 0.5 for patients on subcutaneous human C1-esterase inhibitors, and over 95% of the total healthcare costs consisted of pharmacy costs.

Our finding of continued reductions in ODT/STP costs over time with lanadelumab treatment might reflect a reduced need for these therapies with increased time on effective LTP treatment. Indeed, a study among 32 patients who had received lanadelumab for 4 years found that no attacks were experienced after 146 of 147 medical procedures that have the potential for inducing attacks, despite no use of ODT/STP medications [19]. It may be, therefore, that the need for ODT/STP is mitigated by long-term use of effective LTP.

## Limitations

The results of this study should be interpreted with consideration of the limitations. Although inverse CBPS weighting was used to control for potential confounding of the relationship between treatment type and study outcomes by certain patient characteristics, there were still imbalances in baseline characteristics between the two groups. However, given the small sample sizes for both cohorts, the number of variables included in the regression model used to derive the CBPS was limited and therefore, observed differences in outcomes between cohorts after weighting may have been influenced by other variables not controlled for in these analyses. Future studies with larger sample sizes might consider including additional variables in regression models to derive CBPS for weighting. The small sample size also likely contributed to the fact that the majority of differences failed to reach statistical significance.

The potential exists for misclassification of patient characteristics, lanadelumab or berotralstat use and treatment persistence, as patients were identified through administrative claims data as opposed to medical records, and it was not possible to confirm whether patients took or correctly administered their prescribed medications. As with any claims databases, the MarketScan Research Databases rely on administrative claims data, which are subject to data coding limitations and potential data entry error. However, there is no reason to suspect that errors and misclassification differentially affected lanadelumab- or berotralstat-treated patients.

To preserve sample size due to the shorter time on market for berotralstat, the length of required persistence and follow-up differed between the lanadelumab (18 months) and berotralstat (6 months) cohorts. This has the potential for introducing selection bias, as patients receiving lanadelumab with longer continuous enrolment might have been healthier than those receiving berotralstat. It is also possible that treatment adherence and HCRU and costs among berotralstat-treated patients could differ in subsequent months from that observed during the first 6 months after treatment initiation. However, considering that berotralstat is believed to achieve steady state relatively quickly and there is no labeled indication for reduced dosing frequency, substantive changes in HCRU or costs were not expected after being persistent for 6 months. Additionally, future studies with larger sample sizes should plan to evaluate lanadelumab data from 2020 onwards to improve comparability with berotralstat outcomes.

This study included patients with employer-sponsored commercial, Medicare Supplemental or Medicare Advantage insurance and, as such, the results may not be generalizable to patients with other types of insurance, such as Medicaid or those with no health insurance. In addition, the results cannot be generalized to patients with HAE who are not persistent on lanadelumab or berotralstat treatment for at least 18 or 6 months, respectively. Additionally, rebates are not included in this insurance claims database and therefore were not included in this analysis.

Finally, while costs and resource utilization are important outcomes to assess among patients receiving LTP, they should not be the only consideration when deciding which LTP is most appropriate for a patient. As encouraged by the shared decision-making model, such decisions need to be made on a per patient basis and take individual preferences into consideration. For example, patients with needle phobia might be more compliant with an oral medication even if this is at additional cost; similarly, patients may prefer to maintain more frequent dosing intervals after achieving disease control out of a sense of security or hesitancy to change.

## Conclusion

This descriptive analysis of healthcare insurance data found that after accounting for baseline differences with weighted analysis, patients who initiated lanadelumab had evidence of lower LTP costs over time compared with patients who initiated berotralstat treatment. Future research can help test these findings once more long-term data are available. The labeled indication for a reduction in dosing frequency after a period of attack-free treatment may be a key factor in treatment selection for patients with HAE, given the combination of cost savings and evidence of lower HAE attack frequency observed here among lanadelumab-treated patients. These findings add to prior analyses of lanadelumab patients to highlight the importance of considering both the potential cost savings and unique patient characteristics when making the decision to reduce lanadelumab dosing frequency.

## Summary points

- Hereditary angioedema (HAE) is a rare genetic condition characterized by spontaneous and unpredictable episodes of cutaneous and submucosal swelling.
- Lanadelumab and berotralstat are plasma kallikrein inhibitors that have been approved for long-term prophylaxis for the prevention of attacks in patients with HAE.
- Real-world data comparing the healthcare resource utilization and costs among patients receiving lanadelumab or berotralstat are lacking.
- This retrospective study used administrative healthcare insurance claims data between 2017 and 2023 from the Merative™ MarketScan^®^ Commercial, Medicare and Early View Research Databases.
- A total of 57 patients receiving lanadelumab and 32 receiving berotralstat met the inclusion criteria and were included in inverse probability of treatment weighted analyses.
- A higher proportion of patients treated with berotralstat had an all-cause inpatient admission and emergency room visit than those treated with lanadelumab.
- Total HAE treatment costs and on-demand treatment/short-term prophylaxis costs were lower for lanadelumab-treated patients than those treated with berotralstat.
- Patients with HAE initiating lanadelumab may require less on-demand and supporting HAE treatments and incur lower healthcare costs than patients initiating berotralstat.

## Supplementary Material








