# Supplementary material for: Comparison of real-world healthcare resource utilization and costs among patients with hereditary angioedema on lanadelumab or berotralstat long-term prophylaxis
Source: J Comp Eff Res. 2025 Feb 20;14(4):e240205. doi: 10.57264/cer-2024-0205 (PMC11963383; doi:10.57264/cer-2024-0205)
Supplement: Supplementary file 1 [file cer-14-240205-s1.pdf]

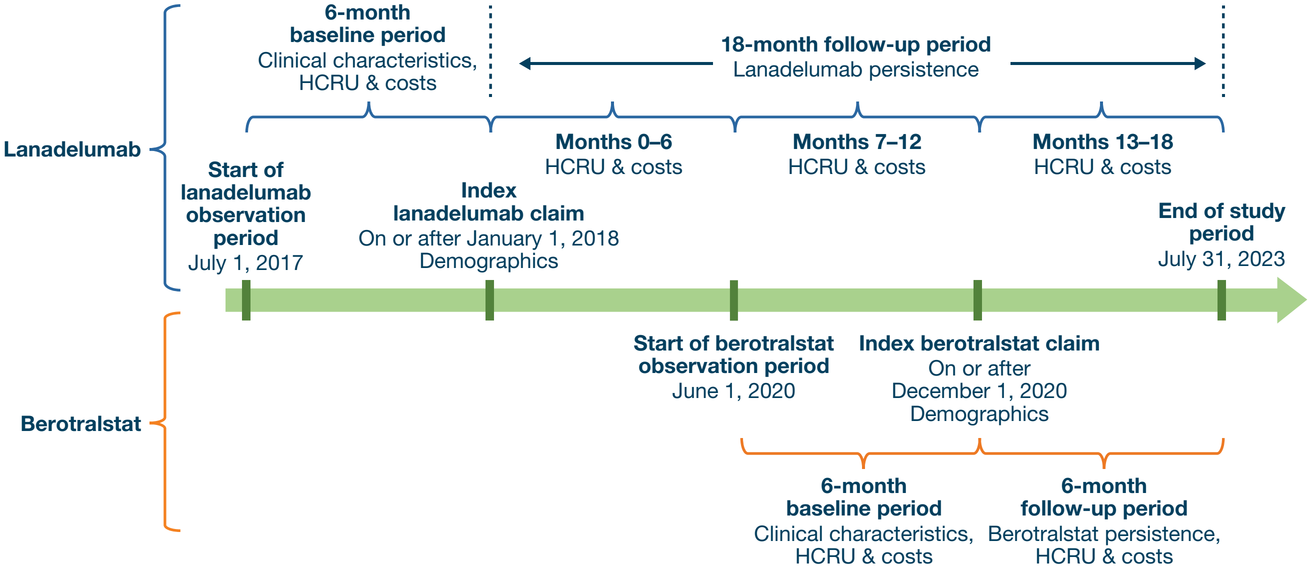

Lanadelumab
6-month baseline period
Clinical characteristics, HCRU & costs
18-month follow-up period
Lanadelumab persistence
Months 0–6
HCRU & costs
Months 7–12
HCRU & costs
Months 13–18
HCRU & costs
Start of lanadelumab observation period
July 1, 2017
Index lanadelumab claim
On or after January 1, 2018
Demographics
End of study period
July 31, 2023
Berotralstat
Start of berotralstat observation period
June 1, 2020
Index berotralstat claim
On or after December 1, 2020
Demographics
6-month baseline period
Clinical characteristics, HCRU & costs
6-month follow-up period
Berotralstat persistence, HCRU & costs

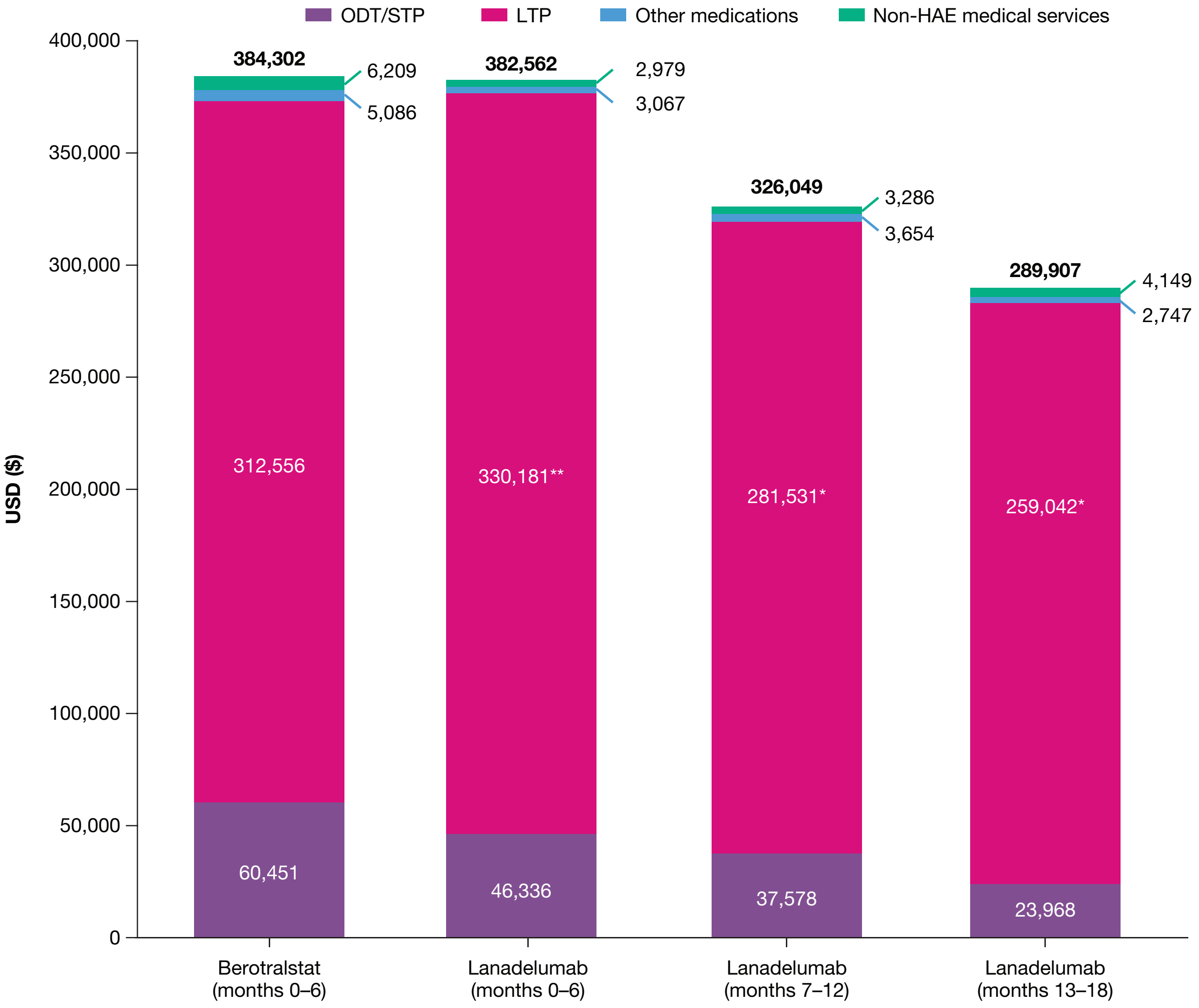

ODT/STP
LTP
Other medications
Non-HAE medical services
USD ($)
400,000
350,000
300,000
250,000
200,000
150,000
100,000
50,000
0
384,302
6,209
5,086
312,556
60,451
382,562
2,979
3,067
330,181**
46,336
326,049
3,286
3,654
281,531*
37,578
289,907
4,149
2,747
259,042*
23,968
Berotralstat (months 0–6)
Lanadelumab (months 0–6)
Lanadelumab (months 7–12)
Lanadelumab (months 13–18)

Figure S1. Study design

HCRU: healthcare resource use.

Figure S2. Total healthcare costs during follow-up (weighted)

*p<0.05 versus berotralstat months 0–6 for lanadelumab and other LTP.

**p<0.001 versus berotralstat months 0–6 for lanadelumab but not other LTP.

HAE: hereditary angioedema; LTP: long-term prophylaxis; ODT: on-demand treatment STP: short-term prophylaxis.
