# Supplementary material for: Comparison of real-world healthcare resource utilization and costs among patients with hereditary angioedema on lanadelumab or berotralstat long-term prophylaxis
Source: J Comp Eff Res. 2025 Feb 20;14(4):e240205. doi: 10.57264/cer-2024-0205 (PMC11963383; doi:10.57264/cer-2024-0205)
Supplement: Supplementary file 2 [file cer-14-240205-s2.docx]

**Table S1.** Patient attrition

|  | **N (%)** |
| --- | --- |
| **Lanadelumab patients** |  |
| At least one medical or pharmacy claim for lanadelumab between 1/1/2018 and 7/31/2023; date of the earliest claim is the index date | 314 (100.0) |
| At least 18 months of continuous enrollment with medical and pharmacy benefits after and including the index date (follow-up) | 128 (40.8) |
| At least 6 months of continuous enrollment with medical and pharmacy benefits before the index date (baseline) | 95 (30.3) |
| Evidence of at least 18 months of lanadelumab persistence | 62 (19.7) |
| No lanadelumab claims during baseline | 62 (19.7) |
| No claims for berotralstat during the study period (7/1/2017–7/31/2023) | 57 (18.2) |
| **Berotralstat patients** |  |
| At least one pharmacy claim for berotralstat between 12/1/2020 and 7/31/2023; date of the earliest claim is the index date | 97 (100.0) |
| At least 6 months of continuous enrollment with medical and pharmacy benefits after and including the index date (follow-up) | 65 (67.0) |
| At least 6 months of continuous enrollment with medical and pharmacy benefits after and including the index date (baseline) | 49 (50.5) |
| No berotralstat claims during the baseline period | 49 (50.5) |
| Evidence of at least 6 months of berotralstat persistence | 32 (33.0) |
