# Supplementary material for: Comparison of real-world healthcare resource utilization and costs among patients with hereditary angioedema on lanadelumab or berotralstat long-term prophylaxis
Source: J Comp Eff Res. 2025 Feb 20;14(4):e240205. doi: 10.57264/cer-2024-0205 (PMC11963383; doi:10.57264/cer-2024-0205)
Supplement: Supplementary file 3 [file cer-14-240205-s3.docx]

**Table S2**. Baseline clinical characteristics

|  | **Berotralstat**  **(n = 32)** | **Lanadelumab unweighted**  **(n = 57)** | **STD** | **Lanadelumab weighted**  **(n = 32)^†^** | **STD** |  |
| --- | --- | --- | --- | --- | --- | --- |
| DCI, mean ± SD | 0.53 ± 0.84 | 0.86 ± 1.82 | 0.23 | 0.58 ± 1.37 | 0.06 |  |
| Inflammatory bowel disease, n (%) |  |  |  |  |  |  |
| Crohn's disease | 1 (3.1) | 0 (0.0) | 0.25 | 0 (0.0) | 0.18 |  |
| Ulcerative colitis | 0 (0.0) | 0 (0.0) | — | 0 (0.0) | — |  |
| HAE triggers/comorbidities n (%) |  |  |  |  |  |  |
| Anxiety or depression | 12 (37.5) | 13 (22.8) | 0.32 | 6.0 (18.8) | 0.39 |  |
| Allergy or anaphylaxis | 13 (40.6) | 24 (42.1) | 0.03 | 13.3 (41.8) | 0.02 |  |
| Anemia | 3 (9.4) | 4 (7.0) | 0.09 | 0.9 (2.8) | 0.23 |  |
| Dental procedure | 0 (0.0) | 0 (0.0) | - | 0 (0.0) | 0.00 |  |
| Diabetes (any) | 1 (3.1) | 5 (8.8) | 0.24 | 3.2 (10.1) | 0.40 |  |
| Fluid and electrolyte disorders | 3 (9.4) | 4 (7.0) | 0.09 | 2.1 (6.6) | 0.10 |  |
| Hypertension | 11 (34.4) | 14 (24.6) | 0.22 | 6.8 (21.5) | 0.27 |  |
| Hypotension | 0 (0.0) | 0 (0.0) | - | 0 (0.0) | 0.00 |  |
| Medications | 5 (15.6) | 9 (15.8) | 0.00 | 6.9 (21.8) | 0.17 |  |
| ACE inhibitor | 0 (0.0) | 1 (1.8) | 0.19 | 0.3 (1.1) | 0.10 |  |
| Hormone replacement therapy | 2 (6.3) | 2 (3.5) | 0.13 | 2.1 (6.6) | 0.02 |  |
| Oral contraceptive | 3 (9.4) | 6 (10.5) | 0.04 | 4.5 (14.1) | 0.16 |  |
| Obesity | 4 (12.5) | 8 (14.0) | 0.05 | 4.7 (14.9) | 0.07 |  |
| Physical trauma or injury | 4 (12.5) | 1 (1.8) | 0.43 | 0.3 (0.8) | 0.35 |  |
| Respiratory infection | 3 (9.4) | 21 (36.8) | 0.69 | 10.6 (33.5) | 0.83 |  |
| Stress | 2 (6.3) | 2 (3.5) | 0.13 | 1.5 (4.6) | 0.07 |  |
| Surgery, broadly defined^‡^ | 9 (28.1) | 8 (14.0) | 0.35 | 3.1 (9.8) | 0.41 |  |
| Surgery, narrowly defined^§^ | 3 (9.4) | 5 (8.8) | 0.02 | 1.4 (4.4) | 0.17 |  |
| HAE attack warning signs, n (%) |  |  |  |  |  |  |
| Abdominal pain | 8 (25.0) | 14 (24.6) | 0.01 | 3.8 (12.1) | 0.30 |  |
| Diarrhea | 3 (9.4) | 5 (8.8) | 0.02 | 1.4 (4.5) | 0.17 |  |
| Dysphonia (hoarse voice) | 0 (0.0) | 1 (1.8) | 0.19 | 0.3 (1.1) | 0.10 |  |
| Fatigue | 0 (0.0) | 4 (7.0) | 0.39 | 2.3 (7.3) | 0.35 |  |
| Fluid retention, edema, swelling | 10 (31.3) | 16 (28.1) | 0.07 | 8.0 (25.2) | 0.13 |  |
| Edema of larynx | 1 (3.1) | 2 (3.5) | 0.02 | 1.6 (4.9) | 0.10 |  |
| Nausea/vomiting | 3 (9.4) | 4 (7.0) | 0.09 | 0.9 (2.8) | 0.23 |  |
| Paresthesia (skin tingling) | 0 (0.0) | 0 (0.0) | - | 0 (0.0) | - |  |
| Pruritis | 1 (3.1) | 0 (0.0) | 0.25 | 0 (0.0) | 0.18 |  |
| Urticaria | 1 (3.1) | 1 (1.8) | 0.09 | 0.3 (0.8) | 0.13 |  |

^†^Patients’ data are weighted by the inverse of their covariate balanced propensity score; post-weighting sample sizes are sums of patients' inverse probability of treatment weights and are therefore not whole numbers for the lanadelumab cohort.

^‡^*CPT* code defined as surgery by the HCUP using a broad definition, or an *ICD-10* procedure code defined by HCUP as a major or minor diagnostic or therapeutic procedure.
^§^*CPT* code defined as surgery by HCUP using a narrow definition, or an *ICD-10* procedure code defined by HCUP as a major diagnostic or therapeutic procedure.

ACE: angiotensin-converting enzyme; *CPT*: *Current Procedural Terminology*; DCI: Deyo Charlson Comorbidity Index; HAE: hereditary angioedema; HCUP = healthcare cost and utilization project; *ICD-10*: *International Classification of Diseases, Tenth Revision*; SD: standard deviation; STD: standardized difference.
