# Supplementary material for: Comparison of real-world healthcare resource utilization and costs among patients with hereditary angioedema on lanadelumab or berotralstat long-term prophylaxis
Source: J Comp Eff Res. 2025 Feb 20;14(4):e240205. doi: 10.57264/cer-2024-0205 (PMC11963383; doi:10.57264/cer-2024-0205)
Supplement: Supplementary file 4 [file cer-14-240205-s4.docx]

**Table S3.** Baseline all-cause healthcare utilization

|  | **Berotralstat**  **(n = 32)** | **Lanadelumab unweighted**  **(n = 57)** | **STD** | **Lanadelumab weighted**  **(n = 32)^†^** | **STD** |
| --- | --- | --- | --- | --- | --- |
| ≥1 inpatient admission, n (%) | 4 (12.5) | 7 (12.3) | 0.01 | 3.2 (10.0) | 0.08 |
| Number of admissions among all patients, mean ± SD | 0.19 ± 0.59 | 0.16 ± 0.45 | 0.06 | 0.12 ± 0.38 | 0.12 |
| ≥1 ER visit, n (%) | 12 (37.5) | 14 (24.6) | 0.28 | 5.4 (17.1) | 0.42 |
| Number of visits among all patients, mean ± SD | 1.5 ± 3.1 | 1.7 ± 5.8 | 0.04 | 0.9 ± 3.9 | 0.18 |
| ≥1 outpatient office visit, n (%) | 32 (100.0) | 55 (96.5) | 0.27 | 31.2 (98.4) | 0.11 |
| Number of visits among all patients, mean ± SD | 6.7 ± 6.3 | 6.6 ± 7.9 | 0.02 | 4.7 ± 5.1 | 0.32 |
| ≥1 other outpatient visit, n (%) | 32 (100.0) | 53 (93.0) | 0.39 | 29.6 (93.3) | 0.33 |
| Number of visits among all patients, mean ± SD | 13.4 ± 15.2 | 13.4 ± 17.7) | 0.00 | 11.7 ± 15.8 | 0.12 |
| ≥1 outpatient prescription claim, n (%) | 32 (100.0) | 56 (98.2) | 0.19 | 31.4 (98.9) | 0.10 |
| Number of claims among all patients, mean ± SD | 18.9 ± 17.2 | 19.2 ± 20.1 | 0.02 | 14.1 ± 14.8 | 0.28 |

^†^Patients' data are weighted by the inverse of their covariate balanced propensity score; post-weighting sample sizes are sums of patients' inverse probability of treatment weights and are therefore not whole numbers for the lanadelumab cohort.

ER: emergency room; SD: standard deviation; STD: standardized difference.
